# Lentinan protects against pancreatic β‐cell failure in chronic ethanol consumption‐induced diabetic mice via enhancing β‐cell antioxidant capacity

**DOI:** 10.1111/jcmm.16529

**Published:** 2021-04-09

**Authors:** Tijun Wu, Jiahui Wang, Yaru Zhang, Yixue Shao, Xirui Li, Yuqing Guo, Wenyu Dong, Lin Wang, Fang Chen, Xiao Han

**Affiliations:** ^1^ Key Laboratory of Human Functional Genomics of Jiangsu Province Nanjing Medical University Nanjing China; ^2^ Key Laboratory of Oral Diseases of Jiangsu Province Nanjing Medical University Nanjing China; ^3^ Institute of Stomatology Nanjing Medical University Nanjing China

**Keywords:** antioxidant activity, chronic ethanol consumption, Lentinan, Nrf‐2 pathway, pancreatic β‐cell failure, T2DM

## Abstract

Chronic ethanol consumption is a well‐established independent risk factor for type 2 diabetes mellitus (T2DM). Recently, increasing studies have confirmed that excessive heavy ethanol exerts direct harmful effect on pancreatic β‐cell mass and function, which may be a mechanism of pancreatic β‐cell failure in T2DM. In this study, we evaluated the effect of Lentinan (LNT), an active ingredient purified from the bodies of Lentinus edodes, on pancreatic β‐cell apoptosis and dysfunction caused by ethanol and the possible mechanisms implicated. Functional studies reveal that LNT attenuates chronic ethanol consumption‐induced impaired glucose metabolism in vivo. In addition, LNT ameliorates chronic ethanol consumption‐induced β‐cell dysfunction, which is characterized by reduced insulin synthesis, defected insulin secretion and increased cell apoptosis. Furthermore, mechanistic assays suggest that LNT enhances β‐cell antioxidant capacity and ameliorates ethanol‐induced oxidative stress by activating Nrf‐2 antioxidant pathway. Our results demonstrated that LNT prevents ethanol‐induced pancreatic β‐cell dysfunction and apoptosis, and therefore may be a potential pharmacological agent for preventing pancreatic β‐cell failure associated with T2DM and stress‐induced diabetes.

## 1 INTRODUCTION

Chronic alcohol consumption is well established as a major risk factor to trigger the development of type 2 diabetes mellitus (T2DM), which is evidenced by impaired glucose metabolism and insulin resistance.^1^, ^2^, ^3^ Due to the key role of pancreatic β‐cell failure in the pathogenesis of T2DM, increasingly more attention has been paid to the understanding of β‐cell damage induced by excessive heavy ethanol in recent years.^4^, ^5^ Evidences exert that ethanol instigates reactive oxygen species (ROS) production and accumulation in β‐cells both in vitro and in vivo.^6^, ^7^ It should be noteworthy that pancreatic β‐cells are more vulnerable to the toxicity of ROS due to a relatively low enzymatic antioxidative defence system.^8^ Undoubtedly, oxidative stress is an important mechanism involved in excessive ethanol exposure‐induced pancreatic β‐cell dysfunction and apoptosis.^6^, ^9^, ^10^, ^11^ Thus, antioxidant agents may be helpful in the reduction of β‐cells damage induced by ethanol, thereby preventing T2DM development stimulated by chronic alcohol ingestion.

The Lentinan (LNT) is plant‐derived natural polyoses (Lentinus edodes) and has been widely accepted as an alternative medicinal supplement with significant anti‐cancer, anti‐viral, anti‐inflammation, immunoregulation, anti‐coagulant, and anti‐tumour effect.^12^, ^13^ Recently, LNT has been proposed to be potent therapeutics for diabetes,^14^ since it has been demonstrated that LNT has protective effects on pancreatic β‐cells against human islet amyloid polypeptide (hIAPP) or streptozotocin‐induced ROS production and cell toxicity.^15^, ^16^ As a natural polysaccharide, LNT has high antioxidant potential by quenching free hydroxyl radicals and superoxide anions and chelating Fe^2+^.^17^ LNT could protect against oxidative damage in various tissues including keratinocytes, cardiomyocytes, gut, boon marrow and so on.^18^, ^19^, ^20^, ^21^, ^22^, ^23^ However, the antioxidant ability for LNT in ethanol‐induced oxidative stress and the subsequent damage of β‐cells have not been investigated yet.

In this study, we designed experiments to investigate the protective effect of LNT on ethanol‐induced β‐cell dysfunction both in vivo and in vitro. Furthermore, we explored the mechanisms underlying this protective effect and found that LNT protected pancreatic islets and β‐cells against ethanol‐induced damage through Nrf‐2 antioxidant activity.

## 2 MATERIALS AND METHODS

### 2.1 Reagents and chemicals

The Lieber‐Decarli regular liquid diet (control diet, 710027; ethanol diet, 710260) was purchased from Dyets (USA). Male C57BL/6 mice were purchased from the Model Animal Research Center of Nanjing University. The mouse insulin ELISA kit was obtained from Ezassay. RPMI‐1640, DMEM, Trizol and Lipofectamine 2000 were purchased from Invitrogen Life Technologies (USA). Foetal bovine serum (FBS) was purchased from GIBCO (USA). LNT (more than 98% purity), ethanol (more than 95% purity) and type V collagenase were obtained from Sigma Aldrich (USA). Antibodies used against NeuroD1, MafA, PDX‐1, PARP‐1, Caspase‐3, Caspase‐9, Nrf‐2, HO‐1 and 4‐HNE were acquired from Cell Signalling Technology (USA). Antibody against insulin was obtained from Santa Cruz (USA). Antibody against GAPDH was purchased from Bioworld (China). The Detergent Compatible (DC) Protein Assay kit was purchased from Bio‐Rad Laboratories (USA). The reverse transcription kit and SYBR Green qPCR Master Mix for mRNA were purchased from Vazyme (China). The TUNEL assay kit was purchased from BOSTER (China). The Annevin V‐FITC/PI apoptosis detection kit was purchased from Vazyme (China). ROS accumulation in the mouse islets and MIN6 cells was analysed using 2′,7′‐dichlorodihydrofluorescein diacetate (DCFH‐DA, Beyotime, China). ROS concentrations in the islets and MIN6 cells were measured using the ROS ELISA kit (Lanpai, China). The superoxide dismutase (SOD) assay kit and glutathione peroxidase (GPx) assay kit were purchased from Millipore (USA). The malondialdehyde (MDA) assay kit was obtained from Nanjing Jiancheng Bioengineering Institute (China). The Luciferase Assay System was obtained from Promega (USA). The firefly luciferase reporter plasmid ARE‐Luc (containing ARE‐binding sites) and small interfering RNA (siRNA) against Nrf‐2 were kind gifts from Professor Dongming Su (Nanjing Medical University, Nanjing, China). The LNT was provided as powder and dissolved in saline (0.9% NaCl) right before in vivo or in vitro administration. All the reagents and chemicals used for animal injection and cell culture were filtered and sterilized before applied in the experiments.

### 2.2 Animal experiments

All animal experiments were carried out in strict accordance with the guidelines and rules formulated by the Animal Care and Ethical Committee of Nanjing Medical University. Mice was randomized into six groups: control diet group (CTRL‐fed) with low dose LNT (3 mg/kg weight) or high dose LNT (10 mg/kg weight) or saline administration, ethanol diet group (EtOH‐fed) with low dose LNT (3 mg/kg weight) or high dose LNT (10 mg/kg weight) or saline administration. To establish chronic ethanol consumption models, mice were housed in cages (four to five per cage), reared under a 12‐hour light/12‐hour dark cycle, and placed on Lieber‐Decarli regular liquid diet containing 5% (v/v) EtOH (36% of calories) or an isocaloric control diet without EtOH (substitution of maltose‐dextrin for EtOH) for at least 8 weeks.^7^, ^24^ Meanwhile, the mice in the LNT administration or saline group were intraperitoneally injected with LNT or equal‐volume saline per day, respectively. The levels of liquid solution intake and body weight gain were followed up almost every week. The daily ethanol intake for EtOH‐fed mice was lower than the lethal dose prescribed by the World Health Organization.^25^

### 2.3 Intraperitoneal Glucose tolerance (IPGTT)

After modelling, the mice were subjected to an intraperitoneal glucose tolerance testing (IPGTT). In brief, after being fasted for 16 hours, the mice were intraperitoneally injected with 2 g/kg body weight of glucose (dissolved in saline). Blood glucose level was then measured from tail vein blood with a glucometer (Roche Diagnostics) and test strips at 0, 5, 15, 30, 60 and 120 minutes after injection. The results for IPGTT were displayed as blood glucose curves and the area under the curve (AUC).

### 2.4 Blood collection and insulin measurement

Mice whole bloods were collected through tail vein at 0, 5, 15, 30 and 60 minutes after glucose injection in IPGTT assay mentioned above. Bloods were then centrifuged at 3000 *g* for 15 minutes to collect serum. Serum insulin were measured by ELISA test kits according to assay instructions. Results were recorded by a micro‐plate reader at 450 nm and the concentration of serum insulin were calculated following a standard curve.

### 2.5 Intraperitoneal Insulin tolerance tests

For intraperitoneal insulin tolerance test (IPITT), the mice were intraperitoneally injected with 1 U/kg body weight of insulin after a 4‐hours fasting, and their blood glucose levels were measured 0, 15, 30, 60 and 120 minutes after injection. The results for IPITT were displayed as a blood glucose curve and the area under the curve (AUC).

### 2.6 Pancreas extraction and immunofluorescence staining

Mice were sacrificed with CO_2_ gas and then the abdominal and thoracic cavity were opened. After totally bleeding by cutting off the right atrial appendage, the complete pancreas was stripped down along the duodenum with surgical forceps. Pancreases obtained from mice were fixed in 10% formalin and then embedded in paraffin for sectioning. The paraffinized sections were heated for 15 minutes at 55°C, deparaffinized (2 × 100% xylene for 5 minutes each, 2 × 100% ethanol for 5 minutes each, 2 × 95% ethanol for 5 minutes each, and 70% ethanol for 5 minutes), and then rinsed in ddH_2_O for 5 minutes. Antigen retrieval was performed by heating the slides at 100°C for 8 minutes in an acidic retrieval solution. The samples were blocked in 3% (w/v) BSA for 15 minutes at RT before incubating at 4°C overnight with primary antibodies against 4‐HNE, Nrf‐2, insulin or HO‐1 diluted in 3% BSA. After being washed, the specimens were incubated in fluorochrome‐conjugated secondary antibody diluted in 3% BSA for 1 hour at RT in the dark. Nuclei were stained with DAPI and then secured with a coverslip. Images were obtained using a laser scanning microscope (Olympus).

### 2.7 Islet perfusion

After equilibrating overnight, 130 islets per group were incubated 1 hour at 37°C in Krebs‐Ringer buffer (KRB) solution with 2 mmol/L glucose. Then, islets were collected in a syringe filter (Millex‐GP; Millipore) for further perfusion. 37°C KRB solution with 2 mmol/L glucose were perfused at 125 µL/min for 15 minutes to equilibrate, then the perfusate were collected per minute for another 6 minutes. After that, 37°C KRB solution with 20 mmol/L glucose were perfused for 25 minutes and the perfusate were collected as previous. Totally, 7‐12 minutes was determined as the first phase insulin secretion while 12‐30 minutes was defined as the second phase of insulin release. The insulin levels of the perfusate were measured by radioimmunoassay (RIA) as previously described.^26^

### 2.8 Glucose/KCl‐stimulated insulin secretion assay

MIN6 cells were pretreated with LNT for 2 hours and then exposed to ethanol for another 48 hours, then glucose‐stimulated insulin secretion assay (GSIS) and KCl‐stimulated insulin secretion assay (KSIS) were performed as previously described.^27^

### 2.9 Cell culture

The mouse pancreatic β‐cell line MIN6 was established as described previously^27^ and was cultured in Dulbecco's Modification of Eagle's Medium (DMEM) containing 15% FBS (Gibco), 10 mmol/L HEPES, 1 mmol/L sodium pyruvate, 100 U/mL penicillin, 100 μg/mL streptavidin and 50 μmol/L β‐mercaptoethanol. Cells were cultured at 37°C in a humidified atmosphere containing 95% air and 5% CO_2_.

### 2.10 Cell viability assay

Cell viability was measured by MTT [3‐(4,5‐dimethyle‐2‐thiazolyl)‐2,5‐diphenyl‐2‐H‐tetrazolium bromide] assay. In short, at least 1 × 10^4^ cells were used for each experiment. MIN6 cells were grown in 96‐well plates. Cells were pretreated with LNT at concentrations of 0, 50, 100, 200 and 400 μg/mL for 2 hours and then exposed to ethanol (60 mmol/L) for an additional 72 hours. Each well was then supplemented with 10 μL MTT and incubated for 3 hours at 37℃. Finally, the formazan precipitate was dissolved in dimethyl‐sulphoxide (Sigma‐Aldrich) and the absorbance was measured at 490 or 570 nm using microplate reader (Perlong, China).

### 2.11 TUNEL staining

Cells were fixed for 0.5 hours in 4% paraformaldehyde. Terminal dUTP nick‐end labelling (TUNEL) was performed with a commercially available kit (BOSTER, China) according to manufacturer's protocol. TUNEL imaging and quantitation of MIN6 cells were observed under laser scanning confocal microscope (FV1200, Olympus, Japan).

### 2.12 Western blot analysis

Cells were lysed with ice‐cold lysis buffer, and protein concentration in the cell lysate was quantified using the DC protein assay kit (Bio‐Rad). After protein content determination using a DC Protein Assay kit, Western blot analysis was performed as described previously.^28^

### 2.13 Flow cytometry assay

MIN6 cells were grown in the wells of 6‐well plates and pre‐treated with LNT for 2 hours, and then exposed to ethanol or LNT for an additional 72 hours. The cells were harvested by trypsinization. Following double staining with FITC‐annexin V and propidium iodide (PI) (Vazyme, China), the cells were analysed using flow cytometry.

### 2.14 Quantitative RT‐PCR analysis

Cellular RNA of β‐cell lines or islets were isolated using Trizol reagent (Invitrogen). cDNA was prepared from RNA using the SYBR Green PCR Master Mix (Vazyme), and quantitative RT‐PCR was performed using a LightCycler480 II Sequence Detection System (Roche) as previously described.^27^ Data were normalized by *Actin*, and mRNA changes were calculated by the comparative ΔCt method. The primer sequences are shown in Table S1.

### 2.15 Analysis of intercellular reactive oxygen species (ROS) levels

Intercellular ROS accumulation was analysed using 2′,7′‐dichlorodihydrofluorescein diacetate (DCFH‐DA, 10 μmol/L; Beyotime, China) as previously described.^29^ The concentrations of ROS in the islets and MIN6 cells were measured using a mouse ROS ELISA kit (Lanpai, China).

### 2.16 Determination of pancreatic antioxidant and oxidative status

The superoxide dismutase (SOD) assay kit and glutathione peroxidase (GPx) assay kit were used to measure pancreatic SOD and GPx activities, respectively. The pancreatic level of malondialdehyde (MDA) was analysed by the kit from Nanjing Jiancheng Bioengineering Institute (China). The assays were performed according to the manufacturer's instructions.

### 2.17 Transient transfection and luciferase reporter assay

Transient transfection with plasmids or siRNAs was performed with Lipofectamine 2000 as previously described.^26^ ARE luciferase activity was assessed in MIN6 cells using the reporter constructs and the Dual‐Glo Luciferase Assay System (Promega) on a TD‐20/20 Luminometer (Turner BioSystems).

### 2.18 Statistical analysis

All of the experiments above were performed independently at least three times. The results are presented as means ± SEM. Statistical difference between groups was determined by Student's *t* test, and comparisons among groups were performed using ANOVA. A *P*‐value of less than .05 indicated statistical significance.

## 3 RESULTS

### 3.1 LNT attenuates chronic alcohol consumption‐induced impaired glucose metabolism

**FIGURE 1:**
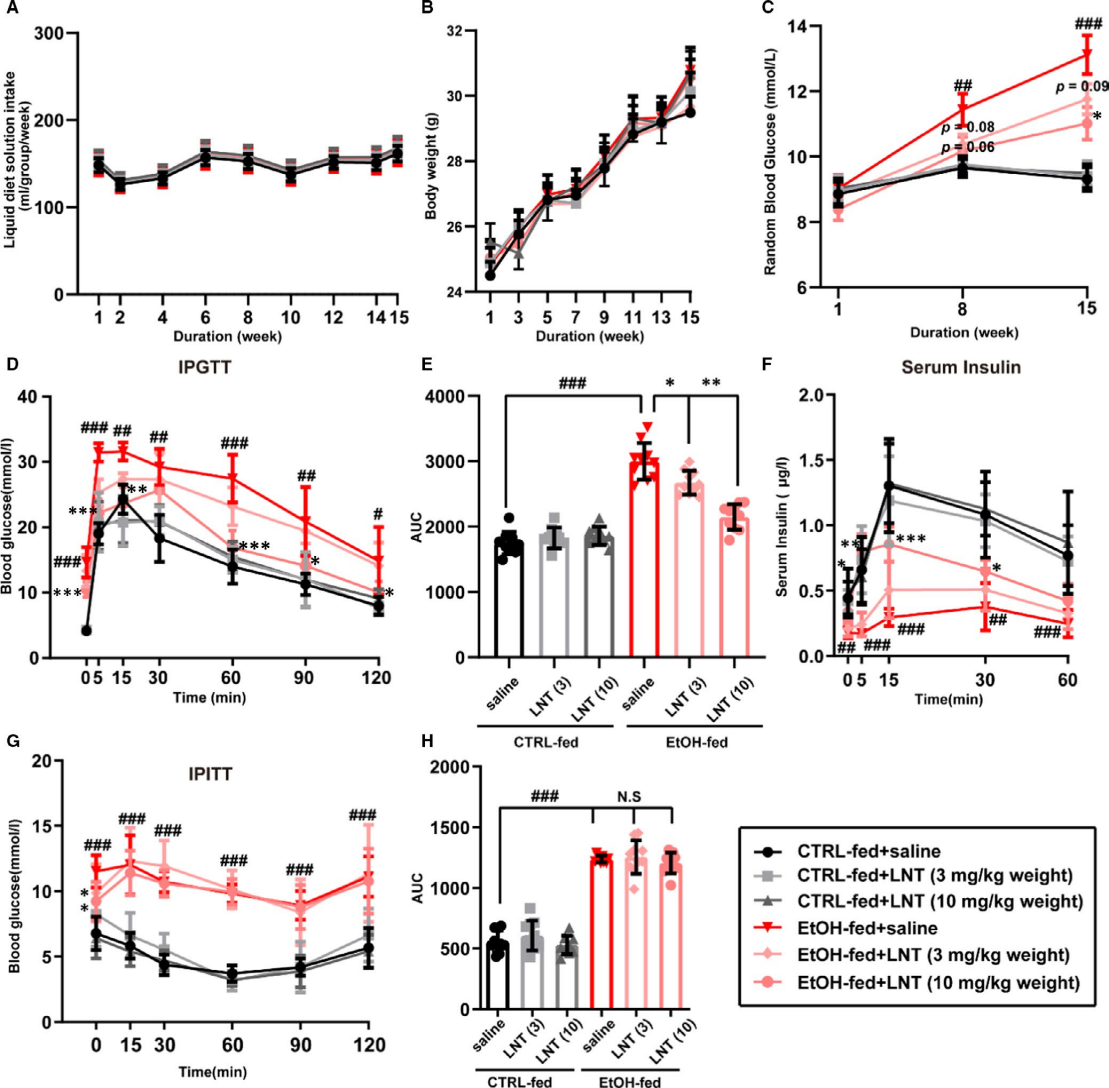
LNT attenuates chronic alcohol consumption‐induced impaired glucose metabolism. A‐B, Liquid diet solution intake and body weight during modelling (n = 10). C, Random blood glucose level was measured in mice 8 and 15 weeks after modelling. D‐E, IPGTT was performed in mice from all six groups (CTRL‐fed with saline or low‐/high‐lose LNT; EtOH‐fed with saline or low‐/high‐lose LNT), and the area under curves (AUC) was calculated in (E). F, Serum insulin levels after glucose stimulation were measured by ELISA for mice from all groups. G‐H, IPITT results for mice from all groups, and the AUC was calculated in (H). Data are presented as mean ± SEM. n = 10 for each group. For C to H, ^#^ *P* < .05, ^##^ *P* < .01, ^###^ *P* < .001 vs. CTRL‐fed + saline group; *P < .05, **P < .01, ***P < .001 vs. EtOH‐fed + saline group

To determine that whether LNT could alleviate impaired glucose metabolism caused by chronic alcohol consumption in vivo, mice was randomized into six groups: control diet group with low‐dose LNT (3 mg/kg weight), high‐dose LNT (10 mg/kg weight) or saline administration, ethanol diet group with low‐dose LNT (3 mg/kg weight), high‐dose LNT (10 mg/kg weight) or saline administration. Mice were placed on a Lieber‐Decarli regular liquid diet (control diet or 5% (v/v) ethanol diet) for more than 8 weeks. Meanwhile, to evaluate the effect of LNT on alcohol‐induced impaired glucose metabolism, the mice from EtOH‐fed group were intraperitoneally injected with LNT daily. Results as shown in Figure 1A‐B, although liquid diet intake and body weight fluctuated during modelling, there was no significant difference between the groups. As expected, random blood glucose in EtOH‐fed group began to rise 8 weeks after modelling. Surprisingly, LNT dose‐dependently resisted hyperglycaemia induced by chronic ethanol intake (Figure 1C). Meanwhile, IPGTT results revealed a marked improvement in the glucose response and serum insulin levels of mice from EtOH‐fed group with LNT administration when compared with those injected with saline, in which high‐lose LNT more effectively alleviated impaired glucose tolerance and insulin secretion (Figure 1D‐F). In addition, LNT had no effect on insulin resistance instigated by ethanol consumption (Figure 1G‐H). Thus, observations above indicated that LNT exerts a robust effect on the treatment of chronic alcohol consumption‐induced impaired glucose metabolism, and LNT may achieve its therapeutic effect through alleviating β‐cell failure induced by ethanol.

### 3.2 LNT ameliorates chronic alcohol consumption‐induced β‐cell dysfunction

**FIGURE 2:**
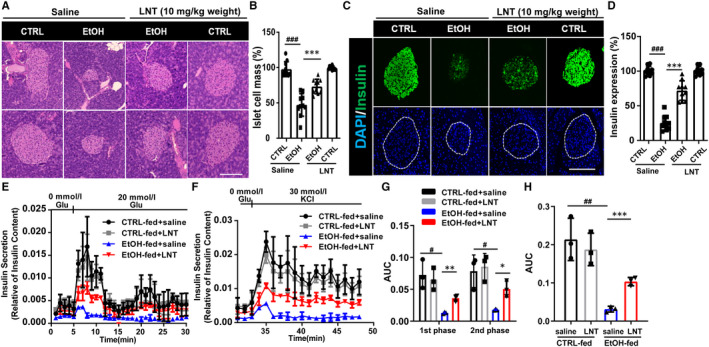
LNT ameliorates chronic alcohol consumption‐induced β‐cell dysfunction. A, The pancreas from CTRL‐fed mice, EtOH‐fed mice with high‐dose (10 mg/kg weight) LNT or saline administration were subjected to haematoxylin and eosin (H&E) staining. Scale bar, 100 μm. CTRL, CTRL‐fed group; EtOH, EtOH‐fed group. B, Islet cell mass in A were quantified, n = 10. C, The pancreas from CTRL‐fed mice, EtOH‐fed mice with high‐dose LNT or saline administration was subjected to immunofluorescence for insulin (green). Scale bar, 100 μm. D, Insulin expression in C was quantified, n = 10. (E,F) Isolated islets were extracted from mice in all four groups (CTRL‐fed, EtOH‐fed with high‐dose LNT or saline administration) during islet perfusion in three separate experiments. G, AUC values for the 1st and 2nd phases of GSIS in islet perfusion in E were calculated. H, AUC values for KSIS in islet perfusion in F were calculated. Data are presented as mean ± SEM. For B, D, G, H, ^#^ *P* < .05, ^##^ *P* < .01, ^###^ *P* < .001 vs. CTRL‐fed + saline group; *P < .05, **P < .01, ***P < .001 vs. EtOH‐fed + saline group

To investigate whether LNT could reverse β‐cell dysfunction induced by chronic alcohol consumption, we extracted the pancreas and islets from those mice for further detection after modelling. LNT significantly alleviated decreased islet cell mass (Figure 2A‐B) and insulin expression (Figure 2C‐D) arose from chronic alcohol intake. Moreover, islet perfusion assay revealed that islets from EtOH‐fed group secreted less insulin both at the first and second phases (Figure 2E,G), which was partially reversed by LNT administration. Also, KCl‐stimulated insulin secretion was obviously improved in islets from EtOH‐fed group with LNT administration (Figure 2F,H). These data demonstrated that LNT ameliorates β‐cell dysfunction, thereby alleviating glucose metabolism disorders stimulated by chronic alcohol consumption.

### 3.3 LNT protects β‐cells against ethanol‐induced impaired insulin secretion and synthesis

**FIGURE 3:**
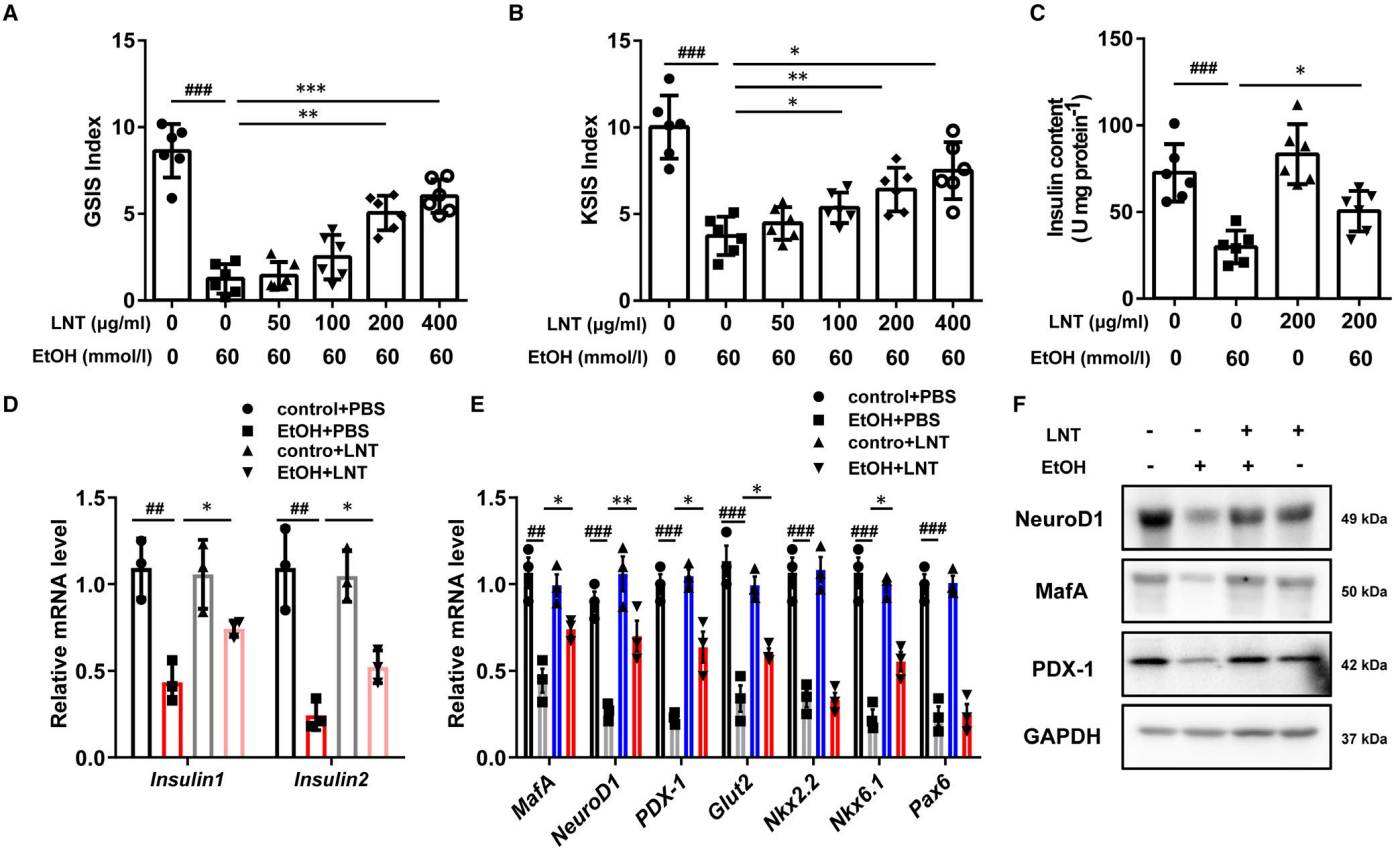
LNT protects β‐cells against ethanol‐induced defected insulin secretion and synthesis. MIN6 cells were pretreated with LNT at different concentrations (0‐400 μg/mL) for 2 h and then exposed to ethanol (60 mmol/L) for another 48 h. GSIS (A) and KSIS (B) indices were calculated (n = 6). C, Insulin content was measured in MIN6 cells treated with ethanol and/or LNT after acidified ethanol extraction (n = 6). D‐E, *Insulin1* and *Insulin2* mRNA levels in MIN6 cells in C were detected via qRT‐PCR assay, other β‐cell important genes mRNA levels were measured in (E). F, The protein levels of NeuroD1, MafA, PDX‐1 in MIN6 cells in C were detected by Western blot assay. GAPDH was used as internal control. Data are presented as mean ± SEM. For A‐E, ^##^ *P* < .01, ^###^ *P* < .001 vs. control + PBS group (LNT:0; EtOH: 0); *P < .05, **P < .01, ***P < .001 vs. EtOH + PBS group (EtOH: 60 mmol/L; LNT: 0)

To investigate whether LNT‐protected β‐cell against ethanol‐induced dysfunction directly, MIN6 cells were pretreated with LNT at different concentrations (0‐400 μg/mL) for 2 hours and then exposed to ethanol (60 mmol/L) for another 48 hours. GSIS and KSIS assay showed that LNT dose dependently reversed ethanol‐induced impaired insulin secretion (Figure 3A‐B), which was similar with those results we obtained in vivo. Moreover, reduction in insulin synthesis caused by ethanol could be also alleviated by LNT treatment (Figure 3C‐D). We further found that ethanol remarkably downregulated β‐cell important genes expression including PDX‐1, NeuroD1, MafA and Glut2 that are essential in the maintenance for normal β‐cell function, while LNT could reverse the reduction in most of these genes (Figure 3E‐F). Taken together, LNT protects β‐cell against ethanol‐induced impaired insulin secretion and synthesis.

### 3.4 LNT prevents ethanol‐induced β‐cells apoptosis

**FIGURE 4:**
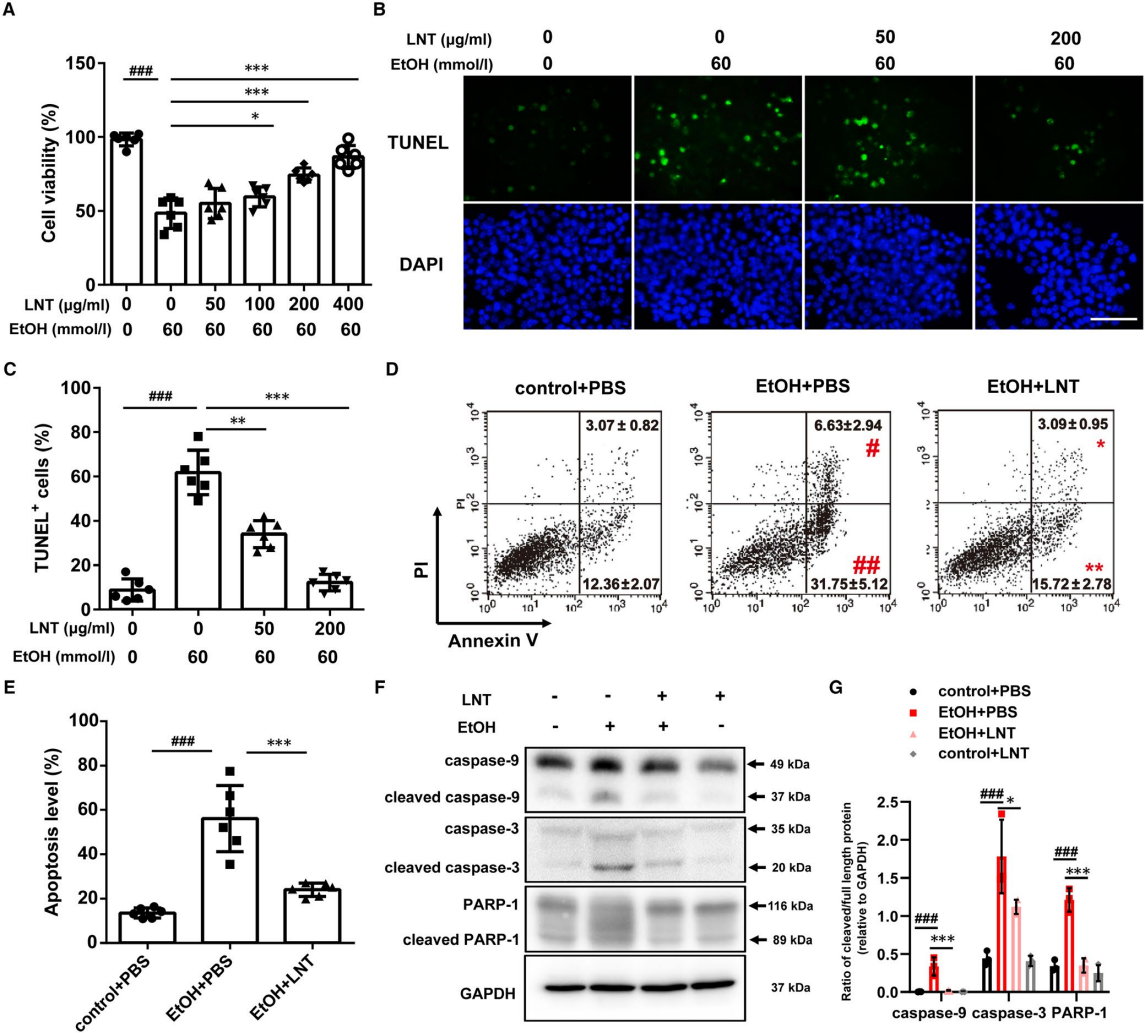
LNT prevents ethanol‐induced β‐cells apoptosis. MIN6 cells were pretreated with LNT at different concentrations (0‐400 μg/mL) for 2 h and then exposed to ethanol (60 mmol/L) for another 72 h. A, Cell viability was evaluated in MIN6 cells using an MTT assay, n = 6. B, TUNEL staining of cellular apoptosis of MIN6 cells. The images of TUNEL positive cells were captured with a confocal laser. Scale bar, 100 μm. C, Quantitative analysis of TUNEL assay results, n = 6. D, Cell apoptosis was detected using flow cytometric assay with Annexin V‐FITC and PI‐staining in MIN6 cells treated with ethanol (60 mmol/L) and/or LNT (200 μg/mL). E, Quantitative analysis of cellular apoptosis detected by flow cytometric measurements, n = 6. F‐G, The protein levels of full length and cleaved PARP‐1, caspase‐3 and caspase‐9 in MIN6 cells treated with ethanol (60 mmol/L) and/or LNT (200 μg/mL). GAPDH was used as internal control. Grey density of cleaved/full length proteins were calculated in (G). Data are presented as mean ± SEM. For A, C, D, E and G, ^#^ *P* < .05, ^##^ *P* < .01, ^###^ *P* < .001 vs. control + PBS group (LNT:0; EtOH: 0); *P < .05, **P < .01, ***P < .001 vs. EtOH + PBS group (EtOH: 60 mmol/L; LNT: 0)

It is reported that excessive alcohol is a significant risk factor in β‐cell apoptosis.^1^, ^2^, ^3^ To examine whether LNT could prevent ethanol‐induced β‐cell apoptosis, MIN6 cells were pretreated with LNT for 2 hours and then exposed to ethanol for an additional 72 hours. MTT assay revealed that LNT remarkedly alleviated ethanol‐induced decreased β‐cell viability in a dose‐dependent manner (Figure 4A). Furthermore, both flow cytometric assay (Figure 4B‐C) and TUNEL assay (Figure 4D‐E) demonstrated that LNT exerted a significant inhibitory effect on ethanol‐induced β‐cell apoptosis. Moreover, Western blot analysis also showed that elevated proteins expression of the cleaved forms of caspase‐3, ‐9, and PARP‐1 in ethanol‐treated MIN6 cells were partially reversed by LNT (Figure 4F‐G). In the above experiments, cell viability and apoptosis were not influenced by LNT concentrations when treated with LNT alone as previously described^15^ (data not shown). Collectively, LNT protects β‐cell from ethanol‐stimulated apoptosis.

### 3.5 LNT enhances β‐cell antioxidant capacity and ameliorates ethanol‐induced oxidative stress

**FIGURE 5:**
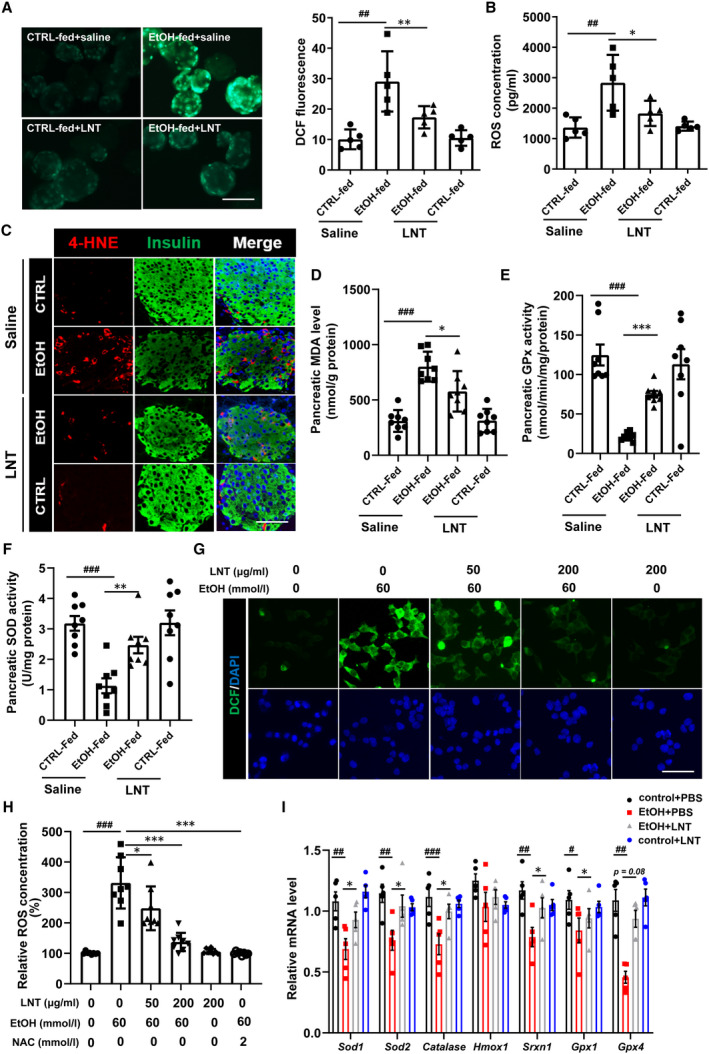
LNT enhances β‐cell antioxidant capacity and ameliorates ethanol‐induced oxidative stress. A, ROS levels determined by DCFH‐DA staining in the islets extracted from mice in CTRL‐fed group, EtOH‐fed group with/without LNT administration. The relative levels of ROS in the islets are presented as DCF fluorescence intensity calculated on the right (n = 5 for each group). Scale bar, 100 μm. B, ROS concentrations were measured by ELISA in islets in A. C, Pancreatic sections from CTRL‐fed group, EtOH‐fed group with/without LNT administration were double labelled for insulin (green) and 4‐HNE (red). Scale bar, 30 μm. D, Pancreatic MDA levels were measured in pancreas extracted from mice in CTRL‐fed group, EtOH‐fed group with/without LNT administration. E‐F, Pancreatic GPx and SOD activity levels were measured by ELISA. G‐H, MIN6 cells were pretreated with LNT at different concentrations (0‐200 μg/mL) for 2 h and then exposed to ethanol (60 mmol/L) for another 36 h. Intracellular ROS levels were detected both by DCFH‐DA staining (G, scale bar, 100 μm) and ELISA (H) in MIN6 cells. NAC (a classical ROS inhibitor) was used as a positive control. I, *Sod1, Sod2, Catalase, Hmox1, Srxn1, Gpx1 and Gpx4* mRNA levels in MIN6 cells treated with ethanol (60 mmol/L) and/or LNT (200 μg/mL) were detected via qRT‐PCR assay. Data are presented as mean ± SEM. For A‐B and D‐F, ^##^ *P* < .01, ^###^ *P* < .001 vs. EtOH‐fed + saline group; * *P* < .05, **P < .01, ***P < .001 vs. EtOH‐fed + saline group. For H‐I, ^#^ *P* < .05, ^##^ *P* < .01, ^###^ *P* < .001 vs. control + PBS group (LNT:0; EtOH: 0); * *P* < .05, **P < .01, ***P < .001 vs. EtOH + PBS group (EtOH: 60 mmol/L; LNT: 0)

Insulin‐producing β‐cells are extremely sensitive to increased ROS accumulation and oxidative stress, which could be severely stimulated by ethanol, which leads to the progressing of β‐cell dysfunction and apoptosis.^30^ As expected, we observed ROS accumulation in islets obtained from EtOH‐fed mice, notably, LNT administration significantly alleviated elevated ROS levels induced by chronic ethanol consumption (Figure 5A‐B). To investigate that whether LNT inhibits ethanol‐stimulated β‐cell oxidative stress, we assessed oxidative stress‐associated markers in the pancreas and islets extracted from EtOH‐fed mice with/without LNT administration. Morphometric analysis for markers of oxidative stress revealed that β cells from mice fed on ethanol diet showed intense cytoplasmic staining for 4‐HNE compared with those fed on control diet, while these effects were reversed by LNT administration (Figure 5C). Consistently, the pancreatic levels of malondialdehyde (MDA) were significantly upregulated in EtOH‐fed mice, which was also lessened under the treatment of LNT (Figure 5D). Meanwhile, the activities of antioxidant enzymes GPx and SOD in pancreatic tissues obtained from EtOH‐fed mice were obviously diminished, while LNT administration dramatically restored activities of those enzymes (Figure 5E‐F).

Similarly, we observed that LNT downregulated intracellular ROS levels in MIN6 cells exposed to ethanol in a dose‐dependent manner (Figure 5G‐H). We further assess the expression levels for antioxidant genes including *Sod1, Sod2, Catalase, Hmox1, Srnx‐1, Gpx1, Gpx4*, and found that a majority of those genes were declined in ethanol‐treated MIN6 cells, while these effects were reversed by LNT (Figure 5I). Together, LNT ameliorates ethanol‐induced oxidative stress in β‐cells.

### 3.6 Nrf‐2 antioxidant pathway is required in LNT treatment on ethanol‐induced β‐cell dysfunction

**FIGURE 6:**
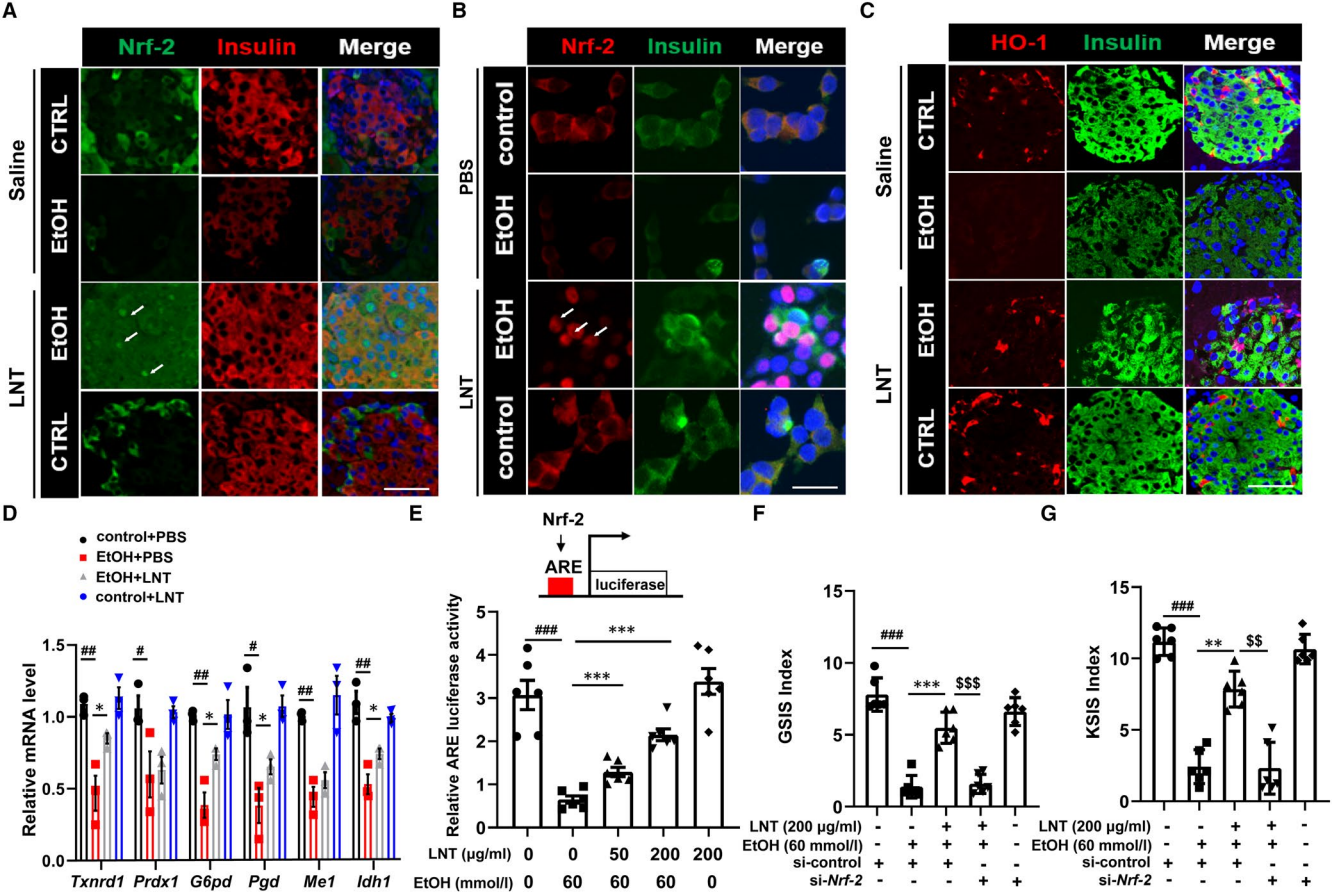
Nrf‐2 pathway is required in LNT treatment on ethanol‐induced β‐cell dysfunction. A, Immunostaining for Nrf‐2 were performed in the pancreatic sections in EtOH‐fed and CTRL‐fed mice, Scale bar, 30 μm. B, Immunostaining for Nrf‐2 were also performed in MIN6 cells treated with ethanol (60 mmol/L) and/or LNT (200 μg/mL). Scale bar, 30 μm. C, Immunostaining for HO‐1 were performed in the pancreatic sections in EtOH‐fed and CTRL‐fed mice, Scale bar, 30 μm. D, *Txnrd1, Prdx1, G6pd, Pgd, Me1, Idh1* mRNA levels in MIN6 cells treated with ethanol (60 mmol/L) and/or LNT (200 μg/mL) were detected via qRT‐PCR assay. E, ARE luciferase activity was measured in MIN6 cells treated with ethanol (60 mmol/L) and/or LNT (0‐200 μg/mL). F‐G, GSIS (F) and KSIS (G) indices were calculated in MIN6 cells as indicated. Data are presented as mean ± SEM. For D‐E, ^#^ *P* < .05, ^##^ *P* < .01, ^###^ *P* < .001 vs. control + PBS group (LNT:0; EtOH: 0); *P < .05, **P < .01, ***P < .001 vs. EtOH + PBS group (EtOH: 60 mmol/L; LNT: 0); For F‐G, ^$$^ *P* < .01, ^$$$^ *P* < .001 vs. EtOH + LNT + si‐control group

The observations above indicated that LNT resists ethanol‐induced β‐cell oxidative stress through maintaining the program of antioxidant genes expression that restores redox balance in response to oxidative stress. Nuclear factor erythroid‐derived‐2‐related‐factor (Nrf‐2) is widely recognized as a prominent regulator of antioxidant enzymes gene expression in β cells.^31^ In the presence of oxidative and xenobiotic stresses, cytoplasmic Nrf‐2 accumulates and translocates into the nucleus, then upregulating antioxidant enzyme expression to conquer oxidative stress.^32^ Thus, we further investigated whether Nrf‐2 antioxidant pathway was involved in enhanced antioxidant capacity in LNT‐treated β‐cells. We observed that cytoplasmic staining for Nrf‐2 was significantly reduced in mice fed on ethanol diet. Interestingly, both nuclear and perinuclear staining for Nrf‐2 was prominent in mice from EtOH‐fed group with LNT administration, while no Nrf‐2 translocation was observed in CTRL‐fed mice injected with LNT or saline (Figure 6A). Similar results were also observed in LNT treated‐MIN6 cells exposed to ethanol (Figure 6B). Heme oxygenase‐1 (HO‐1, Hmox1) is one of Nrf‐2‐dependent antioxidant enzymes, we observed that HO‐1 expression in EtOH‐fed or CTRL‐fed mice was positively correlated with Nrf‐2 (Figure 6C). In addition, we measured other well‐known Nrf‐2 target genes (*Txnrd1, Prdx1, G6pd, Pgd, Me1, Idh1*) and found that LNT could partially reversed their declined expression levels induced by oxidative stress in ethanol‐treated β cells (Figure 6D). Nrf‐2 is a transcription factor that mediates its target genes via acting through antioxidant response elements (ARE) on gene promoters,^33^ as was shown in Figure 6E, ethanol dramatically reduced ARE luciferase activity, which was rebound after LNT treatment dose‐dependently. To further confirm that Nrf‐2 antioxidant pathway participated in the therapeutic effect of LNT, we knock down Nrf‐2 expression and found that depletion of Nrf‐2 diminished the protective effect of LNT on GSIS/KSIS function in ethanol‐exposed MIN6 cells (Figure 6F‐G). Collectively, Nrf‐2 antioxidant pathway mediates LNT treatment on ethanol‐induced β‐cell dysfunction.

## 4 DISCUSSION

Chronic alcohol consumption impairs glucose metabolism and pancreatic β‐cells function, which may trigger the development of type 2 diabetes (T2DM). In the current study, we demonstrated that excessive ethanol exposure results in pancreatic β‐cell failure in vivo and in vitro. Furthermore, our results suggested that LNT, a polysaccharide extracted from Lentinula edodes, has the potential protective effects against ethanol‐induced pancreatic β‐cell impairment by exerting antioxidative stress via Nrf‐2/Keap1/ARE signalling pathways.

Alcohol addiction is a pervasive global problem. Studies have shown that heavy alcohol drinkers are at an elevated risk of developing T2DM relative to non‐drinkers. In addition to ethanol‐mediated peripheral insulin resistance, a central mechanism underlying the deleterious effects of ethanol on pancreatic β‐cell has been intensively reviewed.^5^, ^7^, ^9^, ^24^ Although several studies have explored the therapeutic strategies to protect against ethanol‐induced β‐cell defects, it is a pity that there is still a lack of in vivo verification for the therapeutic effects.^11^, ^34^ In this study, we demonstrated that LNT dose‐dependently alleviated diabetic symptoms of chronic ethanol consumption model mice, manifested as increased random blood glucose, impaired glucose tolerance and insulin secretion. Notably, LNT has no therapeutic effect on excessive ethanol intake‐induced insulin resistance, indicating that LNT could improve glucose metabolism in EtOH‐fed mice only by reversing β‐cell dysfunction. As expected, morphological analysis and islets perfusion assay showed that LNT not only restored β‐cell mass but also enhanced insulin expression, synthesis and biphasic secretion in islets in EtOH‐fed mice. Moreover, we observed that LNT restored β‐cell important genes expression (ie MafA, PDX‐1, NeuroD1, Glut2, etc) and reduced β‐cell apoptosis in MIN6 cells exposed to ethanol in vitro, which should be one mechanism of LNT improving islet function in ethanol‐intake models. In conclusion, LNT rescues impaired β‐cell insulin secretion and β‐cell loss, thereby ameliorates glucose response and glucose metabolism in chronic ethanol intake‐induced diabetic model.

It is well established that ethanol‐induced β‐cell destruction is associated with mitochondrial dysfunction, oxidative stress and increased production of ROS in pancreatic islets.^6^, ^7^, ^35^, ^36^ Consistent with previous studies, we observed ROS accumulation, elevated MDA and 4‐HNE (markers of oxidative stress) in pancreas and islets for EtOH‐fed mice, while LNT administration dramatically reversed those effects. Historically, pancreatic β‐cells are considered to be highly susceptible to oxidative stress since they express very low levels of antioxidant enzymes including catalase (Cat), glutathione peroxidase (Gpx) and superoxide dismutase (Sod).^31^ We observed that chronic ethanol consumption further dampened antioxidant enzymes expression and activities in β‐cells, which was most likely a direct cause for severe oxidative stress. Nevertheless, LNT dramatically restored diminished antioxidant enzymes in EtOH‐fed mice pancreas and MIN6 cells exposed to ethanol, indicating that LNT resists ethanol‐induced β‐cell oxidative stress through maintaining the program of antioxidant gene expression that restores redox balance in response to oxidative stress. Therefore, our studies confirmed the antioxidant capacity for LNT in chronic ethanol intake‐induced β‐cell oxidative stress for the first time. More importantly, LNT may also has therapeutic potential in β‐cell oxidative stress instigated by other diabetogenic factors (ie hyperglycaemia^37^) due to its ability in the maintenance of β‐cell antioxidant system homeostasis.

Nuclear factor erythroid‐derived‐2‐related‐factor (Nrf‐2) serves as a transcriptional factor that orchestrates antioxidant enzymes gene expression and prevents β‐cell damage by counteracting the genotoxic damage induced by ROS accumulation.^38^ Notably, lack of Nrf‐2 allows severe pancreatitis and β‐cell injury after ethanol exposure.^10^ According to our observations, Nrf‐2 expression and its nuclear transcriptional regulation activity were barely detectable in either EtOH‐fed mice islets or MIN6 cells exposed to ethanol, indicating that Nrf‐2 depletion mediates ethanol‐induced β‐cell oxidative stress. Surprisingly, LNT administration not only restored cytoplasmic Nrf‐2 expression but also promoted its entry into the nucleus under oxidative stress status in β‐cells exposed to ethanol. Interestingly, LNT did not lead to altered Nrf‐2 expression and localization in islets or cell lines under physiological status, suggesting that LNT would indirectly maintain Nrf‐2 nuclear expression and transcriptional regulation when oxidative stress occurs. Keap1 (Kelch‐like ECH‐associated protein 1), an adapter protein of Cullin3‐E3 ubiquitin ligase complex, is responsible for Nrf‐2 expression and activation. Under unstressed conditions, Nrf‐2 is constitutively degraded through being bound to Keap1. Upon physiological oxidative stress, Keap1 homodimerization is disrupted that leads to stalled Nrf‐2 degradation, and then cytoplasmatic Nrf‐2 is phosphorylated by PKC and translocates to the nucleus. Nuclear Nrf‐2 ultimately forms a heterodimer with small Maf proteins to bind to antioxidant response elements (ARE) that are localized in regulatory regions in antioxidant genes.^39^ Back to our current studies, it is highly possible that LNT could inhibit excessive Keap1 activity arose from ethanol intake so as to stabilize Nrf‐2 expression and conquer oxidative stress, and we will verify this in our future work. According to our observations, Nrf‐2 pathway activation was essential in LNT treatment for that Nrf‐2 knockdown vanished the protective effect of LNT on ethanol‐induced impaired insulin secretion. Considering that pharmacological activation of the Nrf‐2 pathway in β‐cells has been or currently are being tested in clinical trials for treatment of diabetes and diabetic complications,^31^ more studies are needed to realize the potential for LNT to serve as another effective Nrf‐2 pharmacological activator.

**FIGURE 7:**
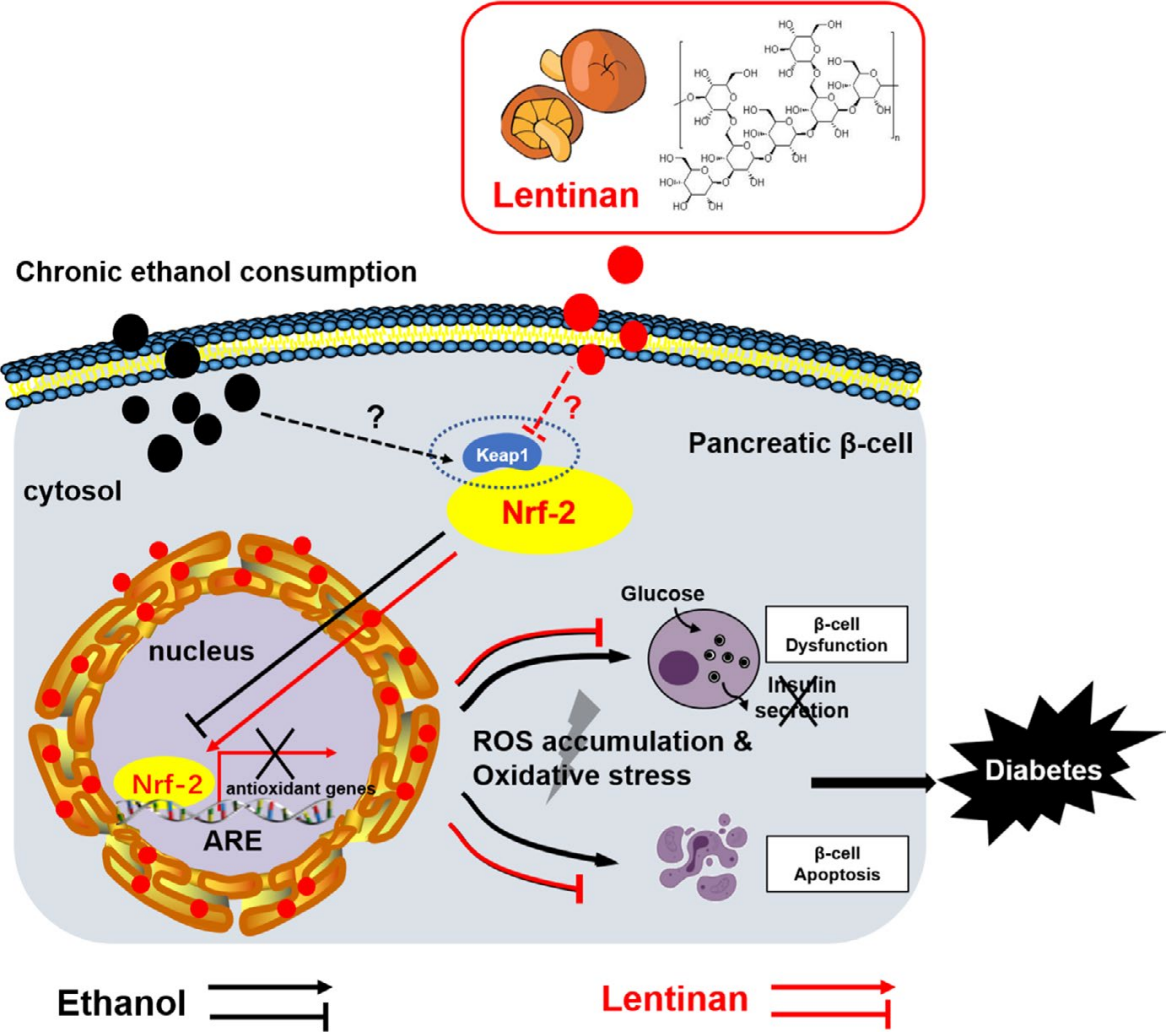
Model of Lentinan‐mediated activation of Nrf‐2/ARE antioxidant pathway in chronic ethanol consumption‐instigated pancreatic β‐cell oxidative stress. Chronic ethanol intake leads to diminished Nrf‐2 expression and its nuclear transcriptional regulation activity (probably due to increased Keap1 activity that degrades Nrf‐2), which induces insufficient antioxidant enzymes activities, ROS accumulation, oxidative stress, and ultimately impaired insulin secretion and β‐cell apoptosis. Lentinan stabilizes both cytoplasmic and nuclear Nrf‐2 expression and conquers ethanol‐instigated β‐cell oxidative stress, thereby improving glucose metabolism and glucose response in chronic ethanol consumption‐induced diabetes

Collectively, the present study provides the first evidence that LNT has the protective effects against ethanol‐induced pancreatic β‐cells damage in vivo and in vitro, the mechanism of which might be ascribed at least partially to the inhibition of ROS generation and oxidative stress through the activation of Nrf‐2/Keap1/ARE antioxidant pathway (Figure 7). These findings imply that LNT would be a useful therapeutic candidate for the treatment of stress‐mediated diabetes.

## CONFLICT OF INTEREST

The authors declare no competing financial interests.

## AUTHOR CONTRIBUTIONS

**Tijun Wu:** Data curation (lead); Funding acquisition (equal); Writing‐original draft (lead). **Jiahui Wang:** Investigation (supporting); Methodology (equal). **Yaru Zhang:** Formal analysis (equal); Investigation (supporting). **Yixue Shao:** Data curation (supporting). **Xirui Li:** Methodology (supporting). **Yuqing Guo:** Software (supporting). **Wenyu Dong:** Writing‐original draft (supporting). **Lin Wang:** Project administration (supporting); Validation (supporting). **Fang Chen:** Formal analysis (lead); Funding acquisition (equal); Resources (lead); Writing‐review & editing (lead). **Xiao Han:** Conceptualization (lead); Funding acquisition (equal); Project administration (equal); Writing‐review & editing (lead).

## Supporting information

Table S1

## Data Availability

The datasets generated during and/or analysed during the current study are available from the corresponding authors upon reasonable request.
