# Supplementary material for: Lentinan protects against pancreatic β‐cell failure in chronic ethanol consumption‐induced diabetic mice via enhancing β‐cell antioxidant capacity
Source: J Cell Mol Med. 2021 Apr 9;25(13):6161–73. doi: 10.1111/jcmm.16529 (PMC8256364; doi:10.1111/jcmm.16529)
Supplement: Supplementary file 1 — Table S1 [file JCMM-25-6161-s001.docx]

**Supplementary Table 1.** Primer sequences for qRT-PCR (5’-3’)

| **Gene** | **Forward** | **Reverse** |
| --- | --- | --- |
| mouse *insulin1* | CACTTCCTACCCCTGCTGG | ACCACAAAGATGCTGTTTGACA |
| mouse *insulin2* | GCTTCTTCTACACACCCATGTC | AGCACTGATCTACAATGCCAC |
| mouse *MafA* | AAGCGGCGCACGCTCAAGAA | GGTCCCGCTCCTTGGCCAGA |
| mouse *NeuroD1* | ATGACCAAATCATACAGCGAGAG | TCTGCCTCGTGTTCCTCGT |
| mouse *PDX-1* | CCCCAGTTTACAAGCTCGCT | CTCGGTTCCATTCGGGAAAGG |
| mouse *Glut2* | TCAGAAGACAAGATCACCGGA | GCTGGTGTGACTGTAAGTGGG |
| mouse *Nkx 6.1* | CTGCACAGTATGGCCGAGATG | CCGGGTTATGTGAGCCCAA |
| mouse *Nkx 2.2* | AAGCATTTCAAAACCGACGGA | CCTCAAATCCACAGATGACCAGA |
| mouse *Pax6* | TGGCAAACAACCTGCCTATG | TGCACGAGTATGAGGAGGTCT |
| mouse *Sod1* | AACCAGTTGTGTTGTCAGGAC | CCACCATGTTTCTTAGAGTGAGG |
| mouse *Sod2* | CAGACCTGCCTTACGACTATGG | CTCGGTGGCGTTGAGATTGTT |
| mouse *Catalase* | AGCGACCAGATGAAGCAGTG | TCCGCTCTCTGTCAAAGTGTG |
| mouse *Hmox1* | AAGCCGAGAATGCTGAGTTCA | GCCGTGTAGATATGGTACAAGGA |
| mouse *Srnx1* | CCCAGGGTGGCGACTACTA | GTGGACCTCACGAGCTTGG |
| mouse *Gpx1* | AGTCCACCGTGTATGCCTTCT | GAGACGCGACATTCTCAATGA |
| mouse *Gpx4* | GATGGAGCCCATTCCTGAACC | CCCTGTACTTATCCAGGCAGA |
| mouse *Txnrd1* | CCCACTTGCCCCAACTGTT | GGGAGTGTCTTGGAGGGAC |
| mouse *Prdx1* | AATGCAAAAATTGGGTATCCTGC | CGTGGGACACACAAAAGTAAAGT |
| mouse *G6pd* | CACAGTGGACGACATCCGAAA | AGCTACATAGGAATTACGGGCAA |
| mouse *Pgd* | ATGGCCCAAGCTGACATTG | GCACAGACCACAAATCCATGAT |
| mouse *Me1* | GTCGTGCATCTCTCACAGAAG | TGAGGGCAGTTGGTTTTATCTTT |
| mouse *Idh1* | ATGCAAGGAGATGAAATGACACG | GCATCACGATTCTCTATGCCTAA |
| mouse *β-actin* | AGGCCAACCGTGAAAAGATG | AGAGCATAGCCCTCGTAGATGG |
